# The Role of Proopiomelanocortin and α-Melanocyte-Stimulating Hormone in the Metabolic Syndrome in Psychiatric Disorders: A Narrative Mini-Review

**DOI:** 10.3389/fpsyt.2019.00834

**Published:** 2019-11-14

**Authors:** Stefan Raue, Dirk Wedekind, Jens Wiltfang, Ulrike Schmidt

**Affiliations:** ^1^Psychotrauma Treatment Unit & RG Stress Modulation of Neurodegeneration, Department of Psychiatry and Psychotherapy, University Medical Center Göttingen (UMG), Göttingen, Germany; ^2^Department of Psychiatry and Psychotherapy, Georg August University, University Medical Center Göttingen (UMG), Göttingen, Germany; ^3^German Center for Neurodegenerative Diseases (DZNE), Göttingen, Germany; ^4^Medical Sciences Department, iBiMED, University of Aveiro, Aveiro, Portugal; ^5^Department of Psychiatry and Neuropsychology, School for Mental Health and Neuroscience (MHeNs), Maastricht University Medical Centre, Maastricht, Netherlands

**Keywords:** posttraumatic stress disorder, schizophrenia, metabolic syndrome, HPA axis, proopiomelanocortin (POMC), PTSD, melanocyte stimulating hormone (MSH)

## Abstract

The metabolic syndrome (MetS) comprises abdominal obesity, preclinical or full diabetes type 2, arterial hypertension, and dyslipidemia and affects a significant proportion of the general population with a remarkably higher prevalence in patients suffering from psychiatric disorders. However, studies exploring the pathogenetic link between MetS and psychiatric diseases are rare. Here, we aim to narrow this gap in knowledge by providing a narrative review on this topic that focuses on two psychiatric diseases, namely on schizophrenia and posttraumatic stress disorder (PTSD) since we assume them to be associated with two different main causalities of MetS: in schizophrenia, MetS evidently develops or aggravates in response to antipsychotic drug treatment while it assumingly develops in response to stress-induced endocrine and/or epigenetic alterations in PTSD. First, we compared the prevalences of MetS and associated pathologies (which we took from the latest meta-analyses) among different psychiatric disorders and were surprised that the prevalences of arterial hypertension and hyperglycemia in PTSD almost doubles those of the other psychiatric disorders. Next, we performed a literature search on the neurobiology of MetS and found numerous articles describing a role for proopiomelanocortin (POMC) in MetS. Thus, we concentrated further analysis on POMC and one of its downstream effector hormones, α-melanocyte-stimulating hormone (α-MSH). We found some evidence for a role of POMC in both PTSD and schizophrenia, in particular in antipsychotic-induced MetS, as well as for α-MSH in schizophrenia, but, surprisingly, no study on α-MSH in PTSD. Taken together, our synopsis reveals, first, a potential interaction between the POMC system and stress in the assumingly at least partially shared pathogenesis of psychiatric disorders and MetS, second, that modulation of the POMC system, in particular of the melanocortin 3 and 4 receptors, might be a promising target for the treatment of MetS and, third, that the DNA methylation status of *POMC* might speculatively be a promising biomarker for MetS in general and, possibly, in particular in the context of stress-related psychiatric conditions such as PTSD. To best of our knowledge, this is the first review on the role of the POMC system in MetS in psychiatric disorders.

## Introduction

### The Metabolic Syndrome in Psychiatric Disorders

Numerous studies have linked psychiatric disorders to a significantly shorter life span. This can result from unawareness of health problems, an unhealthy lifestyle, and particularly from an elevated risk for cardiovascular diseases including one of its major causes, the metabolic syndrome (MetS) (1). Definitions for MetS vary (1), however, common to all of them is that they characterize MetS as a combination of abdominal obesity, preclinical or full diabetes type 2, dyslipidemia, and arterial hypertension. MetS affects one fifth to one third of the general population and goes along with a three-times higher risk for early death (2, 3). Besides the fact that psychopharmacotherapy, in particular second generation antipsychotics (4), can lead to MetS, a wealth of epidemiological studies demonstrates a higher prevalence of MetS in patients suffering from psychiatric diseases. In contrast, studies searching for the molecular mechanisms producing this evident relation of mental disorders and MetS are scarce. The few studies available suggested alterations in the gut microbiome (5), the oxidative/nitrosative stress pathways (6), and in the hypothalamic-pituitary-adrenal (HPA) axis including its major regulator, the glucocorticoid receptor (7, 8), as well as dysfunctions of the immune/inflammatory, melatonin (9), and endocannabinoid systems (10) as shared pathogenic factors of MetS and mental disorders. The most important findings of these publications are that the increased penetration of bacteria across the gut epithelium (leaky gut) may modulate proteins and neurotrophins involved in brain plasticity thereby causing chronic low grade inflammation that further induces MetS (5, 6) that high levels of oxidative and nitrosative stress (IO and NS), metabolic dysregulation and the high comorbidity with the atherogenic components of the MetS classify mood disorders as systemic neuro-IO and NS-metabolic diseases (6), that GR polymorphisms associated with an altered sensitivity to glucocorticoids have been linked to psychiatric diseases and MetS (8) and that adjunctive treatment with melatonin can improve both sleep disturbances and antipsychotics-induced MetS in BPAD patients (9). Moreover, genetic studies which found MetS, schizophrenia, and mood disorders all to share the association with genes encoding for α-ketoglutarate-dependent dioxygenase (*FTO*), an mRNA demethylase, methylenetetrahydrofolate reductase (*MTHFR*), the rate-limiting enzyme in the methyl cycle, and leptin (5, 11, 12) further support the hypothesis of a partially shared pathogenesis of MetS and psychiatric diseases. In addition, genetic variants potentially contributing to the high comorbidity of MetS and schizophrenia were found in the genes of the leptin receptor and the serotonin receptor 2C (*HTR2C*) (12). Furthermore, a number of cardiometabolic disease risk genes, among them those encoding for apolipoprotein E, whose ε4-allele markedly increases the risk for Alzheimer’s dementia (AD) (13), transcription factor cAMP response element-binding protein (CREB), neuroplasticity marker brain-derived neurotrophic factor (BDNF), melatonin receptor 1B (MTNR1B), and proopiomelanocortin (POMC) (11), are known to be associated also with mood disorders. Here, we decided to focus on the latter, i.e., on the POMC-system as a potential common ground of MetS and psychiatric disorders.

### The Role of Proopiomelanocortin in Appetite Regulation

POMC is a precursor peptide constituting the basis for various molecules such as the α-, β-, and γ- melanocyte-stimulating hormones (MSH), ACTH (adrenocorticotropic hormone; one of the major effector hormones of the HPA axis), and β-lipotropin (14–16). All these peptides are part of the anorexigenic system that decreases appetite and food intake. In this system, α-MSH, which is derived from POMC over ACTH, and one of its receptors, the melanocortin 4 receptor (MCR4) (17), seem to play a central role: defects or inhibition of MCR4 have been associated with a higher risk for obesity (18) and both ACTH and MCR4 are known to play a critical role in stress-induced pathologies and associated metabolic side effects. (19–21)

Regulation of cortisol which is released in response to ACTH whose synthesis is, in turn, stimulated by corticotropin releasing hormone (CRH), is essential for homeostasis regulation during stress coping. Notably, an increase or prolongation in cortisol secretion can bring the unwanted effects of obesity and of abnormal changes in fat and glucose metabolism both of which have, in turn, been associated with psychopathological syndromes such as psychosis (14, 22, 23).

The homeostasis of body weight is regulated by a complex system of central and peripheral nervous mechanisms (24). In principal, obesity develops from an imbalance of calorie intake and consumption and is the main risk factor for MetS and thereby also for cardiovascular diseases as a long-term consequence (25). Cross-sectional studies demonstrated that only 30% of obese patients show no evident pathological metabolic alterations (26). The central regulation system of food intake is thought to be located in the arcuate nucleus of the hypothalamus and involves a variety of peptides—for instance, neurons harboring core regulating systems of food intake, i.e., the NPY (neuropeptide Y)/AGRP (Agouti-related protein)- and the POMC-systems, are localized there. They react to blood metabolic peptides or food ingredients. Upon activation of the NPY/AGRP-system, food intake behavior is stimulated while it is inhibited upon activation of the POMC-system (15).

### Metabolic Syndrome in Psychiatric Disorders

We took the prevalence rates of MetS and associated pathologies in posttraumatic stress disorder (PTSD) (27), major depressive disorder (MDD), bipolar affective disorder (BPAD), schizophrenia (28), generalized anxiety disorder (29), alcohol abuse/dependency (30), and neurodegenerative diseases (31) from the latest meta-analyses and summarized them in **Table 1**. Strikingly, PTSD patients have far higher prevalences of arterial hypertension, hyperglycemia, and MetS than all other disorders (**Table 1**). Metabolic diseases interact in different ways with psychopathological syndromes. For example, there is evidence that MDD is a risk factor for overweight and obesity but, in turn, overweight can also be a risk factor for MDD (37). Also, chronic psychosocial stress elevates the risk for MDD and, furthermore, for the vast majority of psychiatric diseases (38) and, of note, also for obesity and metabolic pathologies (39). Apart from this, psychopharmacotherapy strongly impacts on the metabolic system, in particular in schizophrenia (40).

**Table 1:** Overview of the prevalence of MetS and risk factors in several psychiatric disorders.

	PS^1^	BPAD^2^	MDD^3^	PTSD^4^	AUD^5^
**Abdominal obesity** (waist circumference > 102 cm ♂/> 88 cm ♀)	**49.4%**	**48.7%**	**38.0%**	**49.3%**	**38,3%**
**Hyperglycemia** (fasting glucose ≥ 110 mg/dl)	**19.5%**	**11.4%**	**18.8%**	**36.1%**	**14,3%**
**Arterial hypertension** (blood pressure ≥ 130/85 mmHg)	**38.7%**	**47.1%**	**36.7%**	**76.9%**	**46,5%**
**Hypertriglyceridemia** (≥ 150 mg/dl)	**39.3%**	**39.3%**	**30.1%**	**45.9%**	**43,9%**
**Low HDL** (≤ 40 mg/dl ♂/≤ 50 mg/dl ♀)	**42.6%**	**42.1%**	**31.1%**	**46.4%**	**7,6%**
**Smoking**	**54,2%**	**45,5%**	**41,7%**	**40,5%**	**80,7%**
**MetS**	**32.5%**	**37.3%**	**29.7%**	**38.7%**	**21,8%**

Prevalence rates were taken from the following reviews: ^1^ 32; ^2^ 33; ^3^ 34; ^4^ 35; ^5^ 36. PS, paranoid schizophrenia; BPAD, bipolar affective disorder; PTSD, posttraumatic stress disorder; AUD, alcohol use disorder; MetS, metabolic syndrome.

It is highly likely that the leading causes of the elevated prevalence of MetS in PTSD and in schizophrenia differ as MetS has been reported to predominantly result from antipsychotic drug treatment in schizophrenic patients (40) while extreme and/or chronic stress is assumingly a core pathogenetic factor for MetS in PTSD patients. Notably, both stress exposure and antipsychotic medication have been described to interact with the POMC system which we thus regarded as a promising topic for the review at hand.

### Schizophrenia and Antipsychotic-Induced Metabolic Syndrome

Schizophrenia occurs in approximately 1% of the population and goes along with positive symptoms such as delusions and hallucinations and negative symptoms such as emotional numbing and anhedonia (41). Furthermore, schizophrenic patients show social isolation, have fewer medical contacts than patients with other psychiatric disorders, less physical activity, an unhealthy lifestyle, a significantly higher prevalence of nicotine abuse and, moreover, are often non-compliant to medical treatment which results in a high re-hospitalization rate (1, 41–43). Interestingly, there is evidence that the risk for MetS is neither elevated in patients with the first episode of schizophrenia nor in schizophrenic patients without drug treatment. At the beginning of antipsychotic treatment and with increasing numbers of psychotic episodes, the risk of MetS increases up to 35,3%, upon treatment with clozapine even up to 53,8% suggesting a high impact of antipsychotic treatment on the metabolic system (44, 45). Most antipsychotics interact with the metabolic system *via* a stimulation of food intake as well as by elevating the glucose intolerance and lowering insulin sensitivity (46).

In addition, the so-called two-hit hypothesis of schizophrenia suggests the onset of schizophrenia to result from an interplay of genetic and environmental factors such as stress. As mentioned above, the HPA axis plays a central role in stress coping and thus in mental disorders. However, in comparison to other psychiatric diseases, there is so far relatively few research on the role of the HPA axis in schizophrenia (47–49).

### The Role of Proopiomelanocortin and Alpha Melanocyte-Stimulating Hormone in Antipsychotic-Induced Metabolic Syndrome

In summary, the obesity- and MetS- inducing effects of antipsychotic drugs are well described (50, 51). One remaining question is why these side effects do not occur in all patients treated with antipsychotics (52). This motivated the search for related genetic polymorphisms and associated epigenetic alterations that confer the risk for drug-induced metabolic side effects. In this context, MCR4 is one of the candidate target molecules since it has already been described as a risk factor for antipsychotic-induced weight gain (53, 54). Studies that compared the metabolic effects of typical and atypical antipsychotics, which are also called first and second-generation antipsychotics, are very scarce and revealed heterogeneous results. However, there is converging evidence that atypical antipsychotics, in particular olanzapine and clozapine, have a comparably higher potency to elevate serum cholesterol levels (55–57).

The main therapeutic effect of antipsychotic drug treatment results from an inhibition of various central nervous neurotransmitter receptors. For example, clozapine mainly inhibits distinct serotonin receptors (5-HT), histamine receptors (H1), α-adrenergic, and cholinergic receptors (AchM). Olanzapine, another atypical antipsychotic drug known for significant metabolic side effects, has a similar mode of action but a more powerful antidopaminergic effect than clozapine (58). Of note, the neurotransmitters dopamine, histamine, serotonin and their corresponding receptors also play a critical role in the metabolic system. For example, dopamine has an antidiabetic effect that results from the inhibition of prolactin, a luteotropic hormone which impacts on glucose homeostasis. Moreover, there is evidence that histamine controls energy intake *via* stimulation of the H1 receptor that results in an anorectic effect while its long-term inhibition is associated with dyslipidemia (46). The presence of single-nucleotide polymorphisms (SNPs) in serotonin 5HT2A and 5HT2C receptors, which are targeted by several psychotropic drugs and, in addition, have been found associated with obesity and type 2 diabetes, point at a role of serotonin in metabolic homeostasis (59). The neurotransmitters dopamine and serotonin interact directly with the POMC-α-MSH-MC4R system: serotonin stimulates 5HT2c receptor-expressing POMC neurons (15, 60) while dopamine controls dopamine-dependent feeding behavior through its interaction with α-MSH (61).

Furthermore, there is evidence that atypical antipsychotics influence feeding hormones. For example, olanzapine can enhance the effect of ghrelin, an appetite-promoting peptide produced in the gastrointestinal tract and furthermore, causes leptin dysregulation that, in turn, results in increased body weight and diabetes (62, 63). Both leptin and ghrelin are strongly connected to the POMC system (24). In contrast, the effect of atypical antipsychotics on POMC is not yet finally clarified: on the one hand there is evidence that olanzapine increases *POMC* mRNA expression in the rat hypothalamus (64), on the other hand olanzapine was shown to decrease POMC protein concentration in rat brown adipose tissue (65). However, of course, these discrepancies might result from tissue-specificity. In contrast, again another atypical antipsychotic, risperidone, seems to have no effect on *POMC* mRNA expression in the rat hypothalamus (66)—instead, its anti-serotonergic effect seems to play a critical role here (67). In healthy human subjects, olanzapine and quetiapine were found to reduce both blood ACTH and cortisol concentrations (68) and olanzapine was described to increase blood α-MSH levels (67).

The activated MC4R neurons in the hypothalamic system, one of the targets of α-MSH, decrease appetite thereby causing weight loss under physiological conditions. Accordingly, genetic variants in this POMC-MSH system have been associated with antipsychotic drug-induced weight gain (69).

### The Role of Proopiomelanocortin and Alpha Melanocyte-Stimulating Hormone in Posttraumatic Stress Disorder-Associated Metabolic Syndrome

PTSD is also among the psychiatric disorders that have been associated with an elevated prevalence of MetS. In contrast to MetS in schizophrenic patients which mainly seems to be associated with antipsychotic drug treatment, PTSD-associated MetS highly likely results from extreme stress exposure and consecutive alterations in stress hormone systems. According to DSM-5, the PTSD syndrome comprises four main symptoms, i.e., re-aversive re-experiencing and avoidance of trauma-related cues, nervous hyperarousal, and emotional numbing (70). Eating disorders, in particular binge eating disorder, but also anorexia nervosa are common among PTSD patients (71) and might speculatively be interpreted as a dysfunctional stress coping strategy.

In general, psychiatric disorders are known to go along with dysfunctional stress coping on the behavioral and/or the endocrine and molecular level such as alterations in HPA axis regulation. There are two main HPA axis reactivity phenotypes that we previously detected in a population of female PTSD patients subjected to a social stress test: HPA axis responders showed a rapid increase in blood cortisol and ACTH levels in response to stress while the HPA axis non-responder group showed a blunted endocrine HPA axis response despite a marked psychological stress reaction (72). Another research group demonstrated that the proportion of these HPA axis responder types varies among different psychiatric disorders—HPA axis non-responders are particularly frequent in panic disorder patients and particularly rare in populations of individuals free from psychiatric disorders (73). Dysfunctions in stress coping, in particular in regulation of the HPA axis, are one pathogenetic principle known for being involved in the pathogenesis of both MetS and mental disorders (14, 74–76). Of note, POMC, an in particular its DNA methylation (DNAm) status, was found to play a role in HPA axis regulation (77).

Literature on the function of POMC in PTSD is scarce. However, one of the few studies on this topic recently detected that child abuse was associated with an altered epigenetic regulation, i.e., with altered DNAm of the *POMC* gene in blood and saliva samples of a small Tanzanian population (78). Thus, in a future experiment we will look whether our above-mentioned HPA axis responder and non-responder PTSD patient groups and matched controls differ in body weight, blood concentrations of appetite-regulating hormones and in the DNAm status, and expression of leukocyte *POMC* with the aim to, first, replicate the previous findings of (78), and, second, to test whether HPA axis regulation plays a role in the DNAm differences of *POMC* in PTSD and maybe also in the significantly heightened vulnerability for MetS in PTSD (**Table 1**).

As DNAm alterations of *POMC* have also been associated with abuse of various psychotropic drugs/molecules including alcohol in various populations (78), it is likely that the epigenetic regulation of *POMC* may possibly play a role in the heightened prevalence of addictive disorders in PTSD (79). Moreover, DNAm of *POMC* has been associated with body weight regulation, both with underweight (80) and with overweight (81).

Strikingly, we found no study that analyzed the role of α-MSH in PTSD patients. Thus, it will certainly make sense to compare α-MSH blood concentrations in our two HPA axis responder groups of PTSD patients *vs.* healthy controls in future experiments. Interestingly, a study in rats that had been exposed to single prolonged stress (which could be considered an animal model for trauma-related disorders) revealed an improvement of anxiety and depression-like symptoms in response to intranasal treatment with antagonist of one of the receptors of α-MSH, i.e., MCR4 (82).

## Discussion

**Figure 1:**
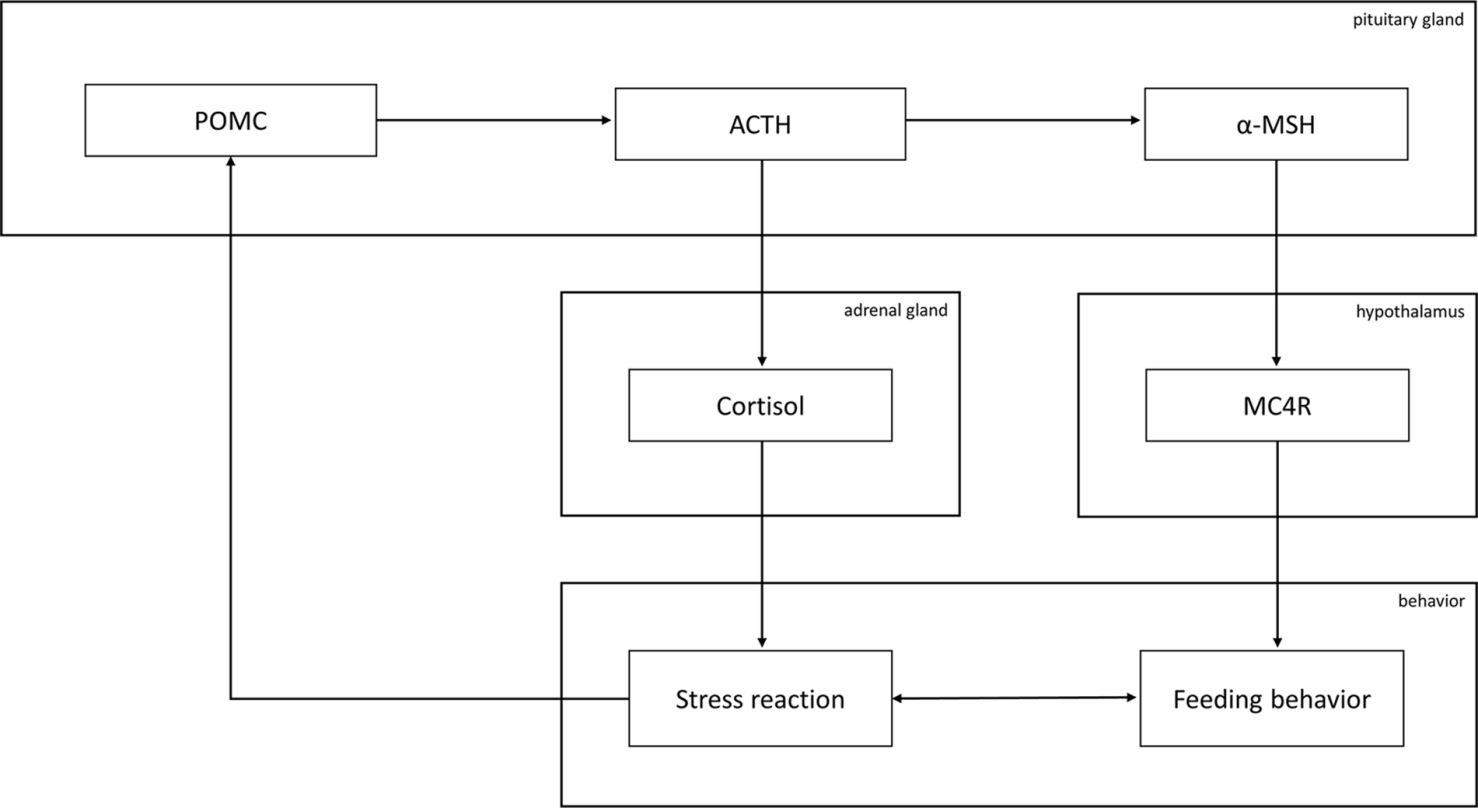
Graphical overview of the involvement of the proopiomelanocortin (POMC) system in feeding behavior. Abbreviations: MC4R, melanocortin receptor 4; α-MSH, α-melanocyte-stimulating hormone; ACTH, adrenocorticotropic hormone. Briefly, ACTH induces the secretion of cortisol, a core mediator of the physiological stress response that, in turn, was reported to influence POMC expression. α-MSH is derived from POMC over ACTH and influences feeding behavior upon binding to MC4R. Feeding behavior and the stress response influence each other.

No clear common mechanisms have emerged between psychiatric disorders and MetS so far. Nevertheless, we found evidence that supports a role for POMC and one of its major downstream effectors, α-MSH, in the pathogenesis of antipsychotic-induced MetS, while there is so far only some evidence for a role of POMC in PTSD and in HPA axis regulation and no study demonstrating the involvement of POMC in PTSD-associated MetS. However, the synopsis of the so far publications at least allows for speculating the latter. Of note, we found no study on α-MSH in PTSD. In general, the HPA axis and the physiological stress response, which have been found to be disturbed in a variety of psychiatry disorders, interact with feeding behavior *via* different pathways, among them POMC-regulated cascades such as α-MSH signaling (**Figure 1**).

The fact that POMC and α-MSH, are associated with the pathogeneses of both MetS and psychiatric disorders such as schizophrenia and presumably also PTSD does not allow the conclusion that these molecules are involved in one and the same pathway in the pathobiology of these disorders—future studies, of which we suggested two in the section above, have to clarify this issue. On the other hand, it is likely that the molecular underpinnings of MetS do not differ between individuals with and without psychiatric disorders, but, nevertheless, it might well be, that there might be an interplay of the pathogenetic pathways of psychiatric and metabolic disorders which might potentiate each other`s pathogenicity.

The overarching aim of many studies searching for molecular pathomechanisms is the identification of novel drug targets. We do not consider POMC *per se* a valuable drug target, mainly because of its involvement in a plethora of body functions which does not allow function-specific targeting, however, the DNAm status of *POMC* might possibly be suited as a (vulnerability) biomarker for MetS in patients with stress-related psychiatric disorders such as PTSD, in particular as it previously has been found associated with body weight regulation (83). In contrast to POMC, interestingly, melanocortin-3 and -4 receptors (MC3R, MC4R) that are activated by α-MSH which results from enzymatic cleavage of POMC by prohormone convertase 1, have already been considered promising targets for anti-obesity therapeutics because of their relative specificity for and their central role in energy homeostasis (84).

In summary, our review reveals the POMC-α-MSH-system to be a promising candidate system for MetS in psychiatric disorders.

## Author Contributions

SR wrote the introductory and the schizophrenia sections as well as parts of the discussion section, performed the literature search except from the PTSD and HPA axis section, created table and figure. DW contributed to the literature search (addiction disorders) and corrected the final version of the manuscript. JW contributed to the literature search (dementia and neurodegenerative disorders) and corrected the final version of the manuscript. US designed the overall structure of the review, designed the literature search strategy, supervised the literature search, performed literature search for the PTSD and HPA axis section, wrote parts of the review (PTSD section, abstract and the discussion section), corrected and framed the entire manuscript including figure and table.

## Funding

We acknowledge support by the Open Access Publication Funds of the Göttingen University.

## Conflict of Interest

JW is supported by an Ilídio Pinho professorship and iBiMED (UID/BIM/04501/2013) and the FCT project PTDC/DTP_PIC/5587/2014 at the University of Aveiro, Portugal. He is member of the Advisory Boards of Abbott, Boehringer Ingelheim, Immungenetics, Lilly, MSD Sharp & Dohme and Roche Pharma. Honoraria Lectures: Arbeitsgemeinschaft für Neuropsychopharmakologie und Pharmakopsychiatrie (AGNP), Actelion, Amgen, CSF-Society, Helios Klinikum Wuppertal, Janssen Cilag, Med Update GmbH, Pfizer, Roche Pharma and Vitos Kurhessen-Bad Emstal. For various projects, however, not for the review at hand, he receives funding, namely the Bundesministerium für Bildung und Forschung (BMBF), Deutsche Forschungsgemeinschaft (DFG) and of the European Union (EU). Patents: PCT/EP 2011 001724 and PCT/EP 2015 052945. US gave Honoria Lectures for Janssen Cilag.

The remaining authors declare that the research was conducted in the absence of any commercial or financial relationships that could be construed as a potential conflict of interest.
